# Effect of the proton irradiation on the thermally activated flux flow in superconducting SmBCO coated conductors

**DOI:** 10.1038/s41598-020-58936-1

**Published:** 2020-02-06

**Authors:** W. J. Choi, D. Ahmad, Y. I. Seo, R. K. Ko, Yong Seung Kwon

**Affiliations:** 1 Department of Emerging Materials Science, DGIST, Daegu, 711-873 Republic of Korea; 2 The Korea Electrotechnology Research Institute (KERI), Changwon, 641-120 Republic of Korea

**Keywords:** Superconducting properties and materials, Condensed-matter physics

## Abstract

We investigate changes in the vortex pinning mechanism caused by proton irradiation through the measurement of the in-plane electrical resistivity for *H*//*c* in a pristine and two proton-irradiated (total doses of 1 × 10^15^ and 1 × 10^16^ cm^−2^) SmBa_2_Cu_3_O_7-δ_ (SmBCO) superconducting tapes. Even though proton irradiation has no effect on the critical temperature (*T*_*c*_), the resulting artificial point defect causes an increase in normal state electrical resistivity. The electrical resistivity data around *T*_*c*_ shows no evidence of a phase transition to the vortex glass state but only broadens with increasing magnetic field due to the vortex depinning in the vortex liquid state. The vortex depinning is well interpreted by a thermally activated flux flow model in which the activation energy shows a nonlinear temperature change ${\boldsymbol{U}}{\boldsymbol{(}}{\boldsymbol{T}},{\boldsymbol{H}}{\boldsymbol{)}}{\boldsymbol{=}}{{\boldsymbol{U}}}_{{\boldsymbol{0}}}{\boldsymbol{(}}{\boldsymbol{H}}{\boldsymbol{)}}{{\boldsymbol{(}}{\bf{1}}-{\boldsymbol{T}}{\boldsymbol{/}}{{\boldsymbol{T}}}_{{\boldsymbol{c}}}{\boldsymbol{)}}}^{{\boldsymbol{q}}}$ (*q* = 2). The field dependence of activation energy shows a ${{\boldsymbol{U}}}_{{\bf{0}}}{\boldsymbol{ \sim }}{{\boldsymbol{H}}}^{-{\boldsymbol{\alpha }}}$ with larger exponents above 4 T. This field dependence is mainly due to correlated disorders in pristine sample and artificially created point defects in irradiated samples. Compared with the vortex pinning due to correlated disorders, the vortex pinning due to the appropriate amount of point defects reduces the magnitude of *U*_o_(*H*) in the low magnetic field region and slowly reduces *U*_o_(*H*) in high magnetic fields.

## Introduction

Superconducting coated-conductor tapes, especially YBa_2_Cu_3_O_7-δ_ (YBCO) tapes, have been dramatically improved over the past decade with excellent superconductivity and easy production processes for electric power applications such as cables, motors, transformers and fault current limiters. Recently, long-length YBCO coated-conductor tapes with good superconductivity are commercially manufactured. This improvement was accomplished by using various vapor deposition methods and various novel substrate and buffer layers^1–8^. However, the potential for operation at elevated temperatures such as liquid nitrogen temperature and high magnetic fields is restricted.

In order to further increase the critical current density (*J*_*c*_) for electric power applications at elevated temperatures, improvement of tape material and improvement of vortex pinning by artificial injection of flux pinning centers have been intensively studied^9,10^. Epitaxial films of YBCO has *J*_*c*_ of over 1 × 10^6^ A/cm^2^ at 77 K and zero-field, which has great applicability. However, *J*_*c*_ falls off in the magnetic field as well as drops much in the form of thin films, bulks, tapes and wires suitable for the application^11^. Recently discovered *Re*BCO (*Re*: other rare-earth) compounds promise smaller field dependence of *J*_*c*_ at higher temperatures because of the higher irreversible field^9^. To improve the in-field critical current, there is a tendency to prefer *Re*BCO instead of YBCO in the fabrication of high-*T*_*c*_ superconductors (HTSCs). For instance, one reason for this is that the substitution of Sm instead of Y results in a higher *T*_*c*_ and a larger *J*_*c*_ at higher temperatures^11,12^.

Vortex pinning in HTSCs continues to be of great interest because of its technological impact on applications. Irradiation of particles with high energy, which can control the number, type, and size of defects, has been extensively used to investigate fundamental interactions between vortices and defects. Point defects induced by electron and proton irradiations^13–16^, cascade defects induced by neutron irradiation^17^, and amorphous track-type columnar defects generated by high energy heavy metal irradiation^18,19^ are artificially induced defects studied so far. These defects have been used to study the relationship between pinning defects and *J*_*c*_. Columnar defects and cascade defects exhibit excellent pinning capability, thereby increasing irreversibility to higher temperatures and increasing *J*_*c*_ at high temperatures and high magnetic fields^18,19^. On the other hand, the point defects have a lower pinning capacity than these defects, but the appropriate plastic deformation and entanglement of the vortex at high temperatures will not only enhance *J*_*c*_, but also give rise to various magnetic field dependence of *J*_*c*_^20^.

In this paper, in order to study the vortex flux pinning associated with higher *J*_*c*_ in SmBCO tapes with superior superconductivity than YBCO tapes, we measured the in-plane electrical resistivity under magnetic field before and after proton-irradiation. Especially, we studied the difference between the vortex pinning mechanisms due to point defects induced by the proton-irradiation and due to inherent defects inevitably formed during specimen preparation. Due to the vortex depinning, broadening of electrical resistivity near *T*_*c*_ in magnetic fields was observed, which was well interpreted by the thermally activated flux flow theory.

## Results and Discussion

**Figure 1:**
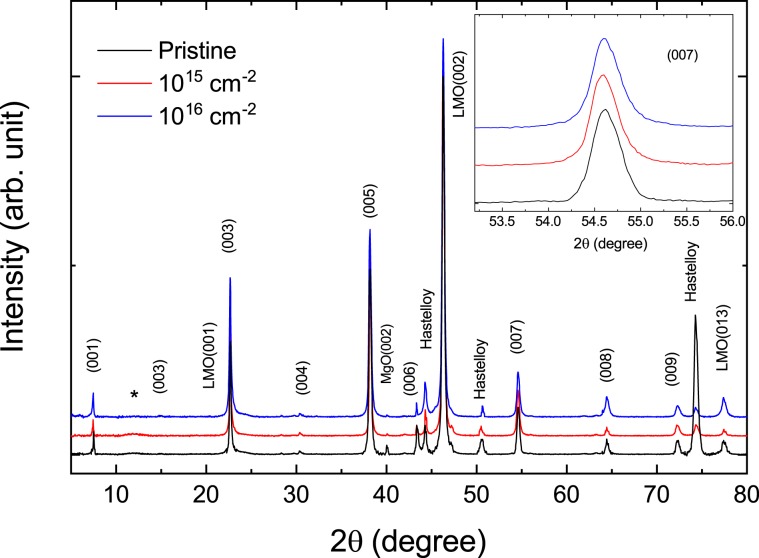
X-ray diffraction (XRD) results on the Bragg-Brentano geometry for pristine, 10^15^ and 10^16^ cm^−2^ proton-irradiated samples. Inset is an enlarged graph of the (0 0 7) peak.

As shown in Fig. 1 for the X-ray diffraction (XRD) results, only (0 0 *l*) peaks diffracted from SmBCO film were observed for the pristine sample except for several peaks of Hastelloy, LMO and MgO used as substrates, indicating that the SmBCO film is *c*-axis-oriented. In the proton-irradiated samples, not only the (00 *l*) peaks observed in the pristine sample but also weak and broad peak (marked with an asterisk) around 12° were observed. This is probably due to a local distortion of the lattice caused by proton irradiation. In particular, the peaks from Hastelloy near 44.3°, 50.5° and 74.3° in the pristine sample increased strongly when compared to those of other samples. This means that there are places where the thickness of SmBCO is not local uniform. Note that XRD experiments on the pristine sample and the two proton-irradiated samples were performed in different parts of the fabricated film. In the inset, (007) peaks look weakly asymmetric. This is likely due to defects and inhomogeneities inevitably occurring during film growth. Nevertheless, the full width at half maximum (FWHM) of the peak in the three samples is 0.35° with almost no difference. The *c*-axis lattice constant calculated from the peaks observed in these three samples was almost unchanged to 11.699 ± 0.001 Å.

**Figure 2:**
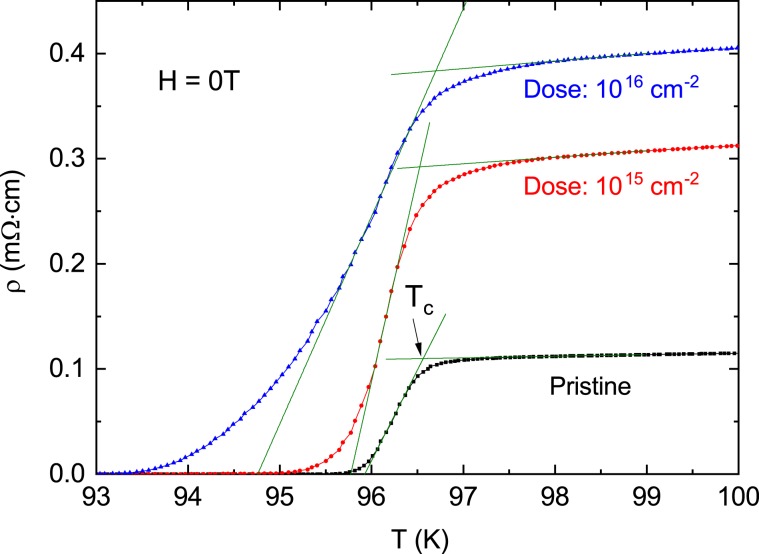
Temperature dependence of electrical resistivity ρ(*T*) near *T*_*c*_ at *H* = 0 for pristine, 10^15^ and 10^16^ cm^−2^ proton-irradiated samples. Green solid lines are lines extrapolated to determine *T*_*c*_.

The electrical resistivity in the vicinity of *T*_*c*_ at *H* = 0 were measured for a pristine sample and two proton-irradiated samples with the dose of 10^15^ and 10^16^ cm^−2^ and plotted in Fig. 2. The magnitude of the electrical resistivity in the normal state just above *T*_*c*_ is larger in the proton-irradiated samples compared to the pristine sample, indicating that quasiparticles are scattered more frequently by irradiation-induced point defects. The superconducting transition temperature *T*_*c*_(0) was determined by the point where the extrapolated straight line near the midpoint of the electrical resistivity jump meets the linearly extrapolated straight line between 98 and 100 K in the normal state. *T*_*c*_(0) is evaluated to be 96.63, 96.55, and 96.70 K (with an error of 0.1 K) for the pristine sample and two proton-irradiated samples with the dose of 10^15^ and 10^16^ cm^−2^, respectively. That is, *T*_*c*_(0) hardly changed within error range in the samples before and after the proton irradiation. Similar behavior was observed in the previously reported proton-irradiated YBCO^21^. On the other hand, when the temperature width, *ΔT*_*c*_, of the superconducting transition is defined as the temperature difference between 0.9·ρ(*T*_*c*_(0)) and 0.1·ρ(*T*_*c*_(0)), it is estimated to be *ΔT*_*c*_ = 0.5, 0.9 and 2.2 K in pristine sample and proton irradiated samples with the dose of 10^15^ and 10^16^ cm^−2^, respectively; an increase in the total dose of irradiated-protons resulted in an increase in the temperature width. From these results, it can be seen that the local scattering between the Cooper pair and the point defects generated by the proton irradiation caused a change in the width of *T*_*c*_ but did not affect *T*_*c*_.

**Figure 3:**
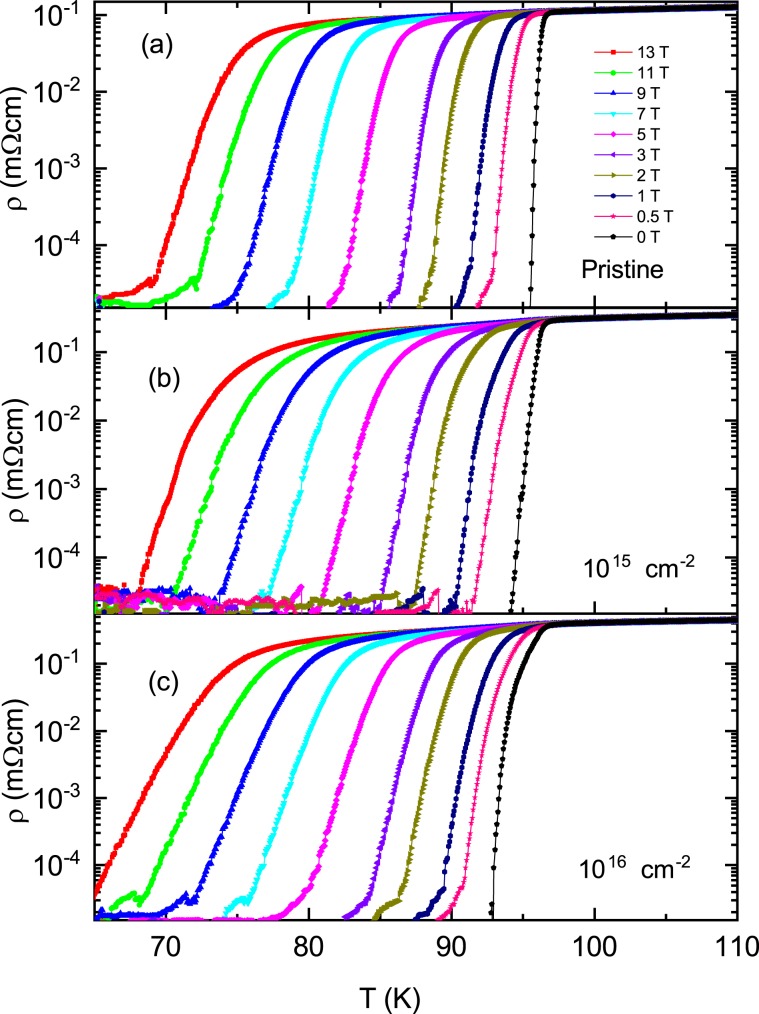
Different color symbols show the in-plane electrical resistivity ρ(*T*) data measured in the magnetic field direction of *H*//*c*-axis in the pristine (**a**), 10^15^ (**b**) and 10^16^ (**c**) cm^−2^ proton-irradiated samples.

**Figure 4:**
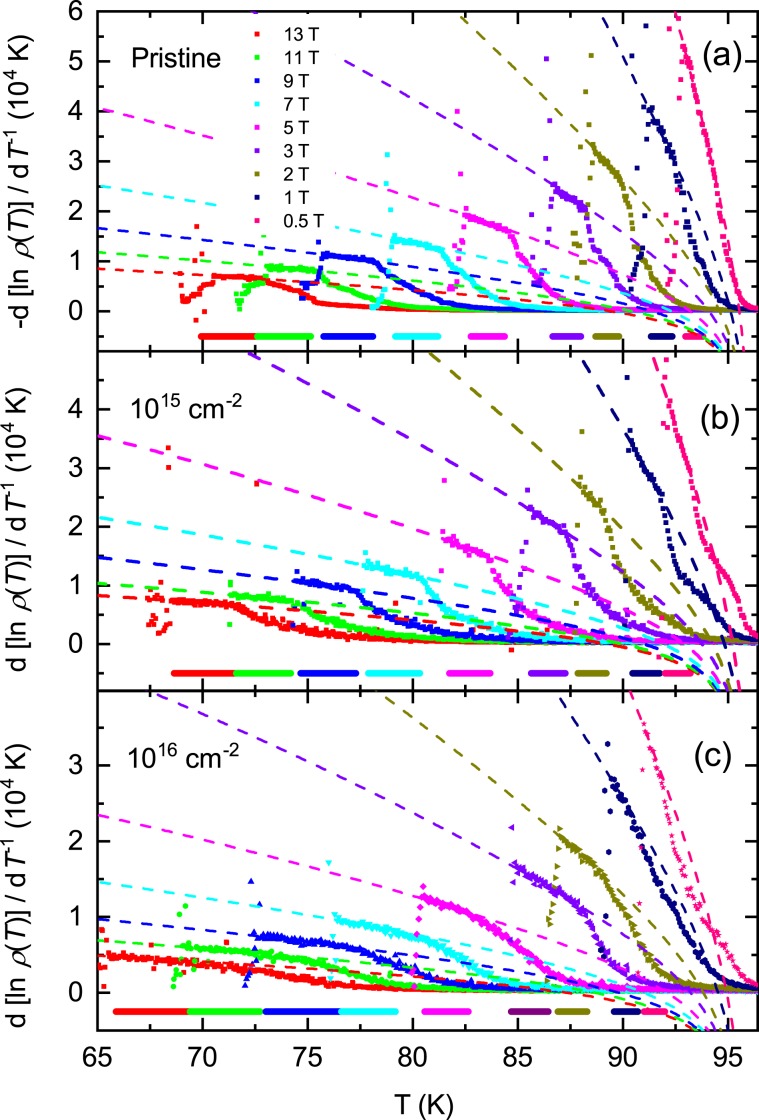
The different color symbols and the dashed-lines show experimental data of $-\partial \,\mathrm{ln}\,\rho /\partial {T}^{-1}$ and the data fitted by Eq. (4) in main text in different magnetic fields for pristine (**a**), 10^15^ (**b**) and 10^16^ (**c**) cm^−2^ proton-irradiated samples, respectively. In (**a**–**c**), the thick horizontal lines of different colors show the TAFF area corresponding to different magnetic fields.

**Figure 5:**
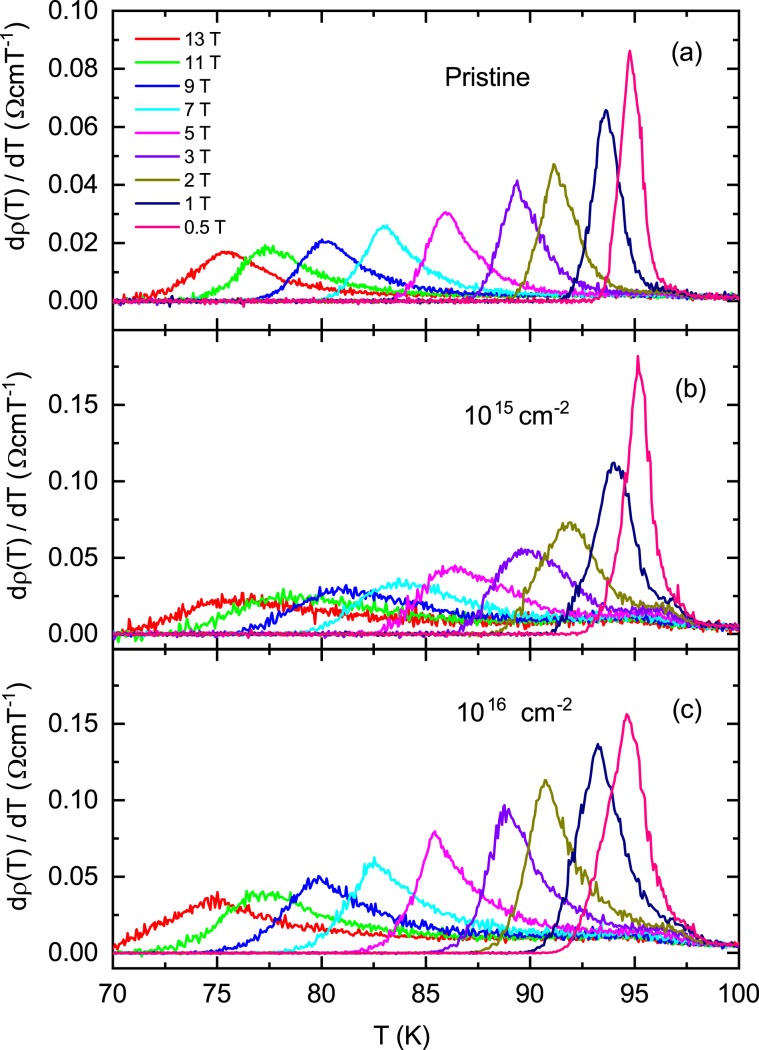
Different color lines show the temperature differential data ${\rm{d}}\rho (T)/{\rm{d}}T$ of in-plane electrical resistivity ρ (*T*) measured in the magnetic field direction of *H*//*c*-axis for the pristine (**a**), 10^15^ (**b**) and 10^16^ (**c**) cm^−2^ proton-irradiated samples.

**Figure 6:**
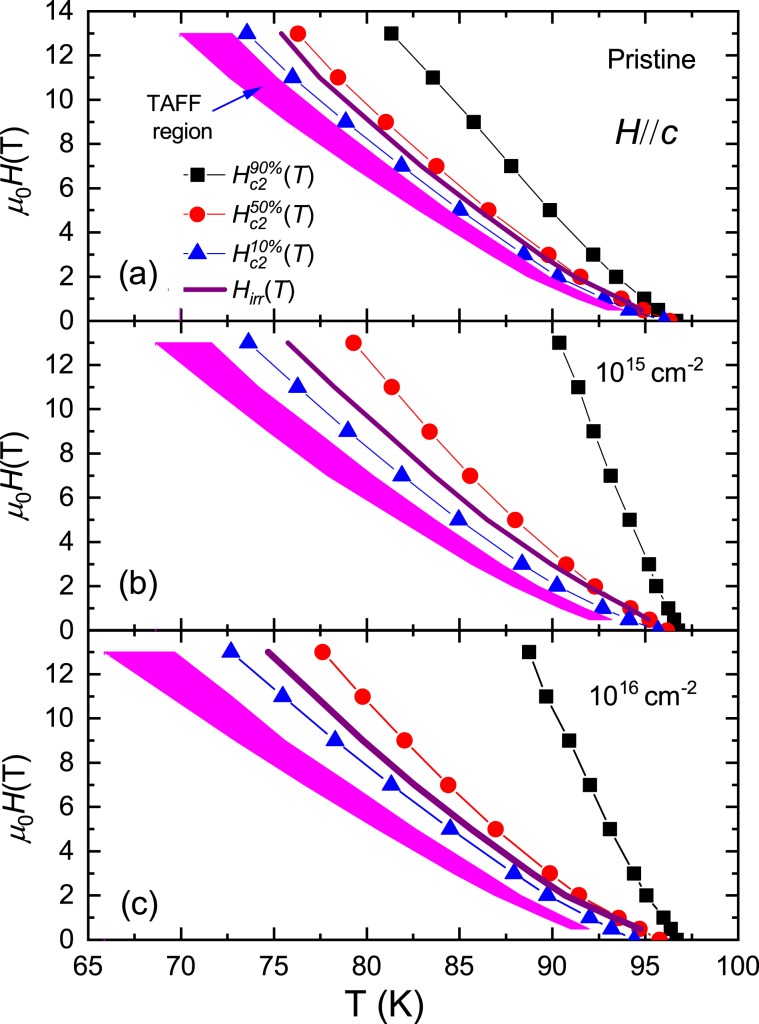
Upper critical fields $({H}_{c2})$, irreversibility field *(H*_*irr*_) and TAFF temperature region decided by the in-plane electrical resistivity data ρ(*T*) for pristine (**a**), 10^15^ (**b**) and 10^16^ (**c**) cm^−2^ proton-irradiated samples. ${H}_{c2}$ curves are determined from the temperatures corresponding to 90, 50 and 10% of the normal resistivity magnitude at *T*_*c*_ obtained by the method described in the text.

The in-plane electrical resistivity ρ(*T*) for *H*//*c*-axis in a pristine sample and two proton irradiated samples with the dose of 10^15^ and 10^16^ cm^−2^ are shown in Fig. 3(a–c), respectively, on a semi-logarithm scale. As the magnetic field increases, the width of the electrical resistivity near *T*_*c*_ gradually widens. Below *T*_*c*_, discontinuous jumps by first-order liquid-to-solid phase transitions observed in clean YBCO single crystals^21^ were not observed in the SmBCO film, indicating that the SmBCO film has inevitable defects during film growth. Then the vortex glass phase in the SmBCO film is expected to be observed below *T*_*c*_, but within the measurement error of the electrical resistivity measurement it was not observed, which will be explained in detail later in the discussion of Fig. 4. It is unknown here whether the vortex glass phase actually exists, but if it does exist it will be observed in the lower temperature range. Therefore, the broadening observed in magnetic fields is definitely due to the thermally activated depinning of the vortex in the vortex liquid phase. The vortex liquid state is divided into the pinned vortex liquid state and the unpinned vortex liquid state^22,23^. To distinguish between the two states, we differentiated each electrical resistivity by temperature, ${\rm{d}}\rho (T)/{\rm{d}}T$, and plotted it as a function of temperature in Fig. 5(a–c). In all three samples, the data at each magnetic field shows a peak at *T*_k_(*B*), and the peak shifts towards lower temperatures as the magnetic field increases. The temperature range above *T*_k_(*B*) is due to the unpinned vortex liquid phase and the temperature area below *T*_k_(*B*) is due to the pinned vortex phase^22,23^. Thus the irreversibility field, *H*_*irr*_ (*T*), at each temperature can be determined from *T*_k_(*B*) and is plotted in Fig. 6(a–c) for a pristine sample and two proton irradiated samples with the dose of 10^15^ and 10^16^ cm^−2^, respectively. *H*_*irr*_ (*T*) did not make a big difference in the three samples. The determined *H*_*irr*_ (*T*) is similar to the previously reported SmBCO coated conductor film but is about 3 T higher than that of commercial YBCO coated conductors^6^. In high temperatures ρ(*T*) gradually decreases towards *T*_*k*_ due to the unpinned flux flow. On the other hand, in the low temperature region ρ(*T*) increases exponentially, that is, ${\rm{\rho }}(T)\propto \exp (-U/T)$ towards *T*_*k*_ due to the pinned flux flow.

From Fig. 3, the higher critical field ${H}_{c2}$ was also determined by the following way: At each field, we first obtain ${T}_{c}(H)$ and ${\rm{\rho }}({T}_{c},H)$ from the electrical resistivity in the same way as in Fig. 2. Then, the temperature corresponding to the value of $0.9\cdot {\rm{\rho }}({T}_{c},\,H)$, $0.5\cdot {\rm{\rho }}({T}_{c},\,H)$ and $0.1\cdot {\rm{\rho }}({T}_{c},\,H)$ was determined from the electrical resistivity data. The upper critical fields ${H}_{c2}$ determined by the 90, 50 and 10% criterions are plotted for the pristine sample and the proton irradiated samples with the dose of 10^15^ and 10^16^ cm^−2^ in Fig. 6(a–c), respectively. The curves of ${H}_{c2}$ for three samples exhibit a slightly upward curvature in low magnetic fields. Similar behavior was reported and discussed in YBCO superconductors^24^ and was attributed to the weak links of the proximity type between superconducting grains. The slope of ${H}_{c2}$ obtained by 90% criterion increases rapidly with proton irradiation. The *H*-*T* area, obtained from 10% and 50% criterions, is very sensitive to flux motions. To determine the maximum *H*_*c*2_(0) from which the effects of the flux motion and the upward bending in low fields are removed, we used the Werthamer-Helfand-Hohenberg (WHH) expression (the orbital limited higher critical field ${H}_{c2}^{orb}(0)=-\,0.693{T}_{c}{({\rm{d}}{H}_{c2}/{\rm{d}}T)}_{{T}_{c}}$) in magnetic fields above 3 T for 90% criterion. As a result, ${H}_{c2}^{orb}(0)$ is evaluated to be 62.3, 140.3 and 118.1 T for a pristine sample and two proton irradiated samples with the dose of 10^15^ and 10^16^ cm^−2^, respectively. *H*_*c*2_(0) in the pristine sample was about half of that of the YBCO film, but *H*_*c*2_(0) in irradiated samples are larger than that of the YBCO film^24,25^. The in-plane Ginzburg-Landau coherence length is obtained by the formula of ${\xi }_{GL}^{in\,plane}(0)={({\varphi }_{0}/2\pi {H}_{c2}^{c}(0))}^{1/2}$; the coherence lengths are evaluated to be 23.0, 15.3 and 16.7 Å for the pristine and the irradiated samples with the dose of 10^15^ and 10^16^ cm^−2^, respectively. The decrease in coherence length to about 50% in the proton-irradiated samples compared to the pristine sample is likely due to the reduction in the mean free path caused by scattering of quasiparticles with many point defects resulting from proton irradiation. But this does not seem to be simple: Judging from the results of the normal electrical resistivity (Fig. 2), the mean free path in the sample with the proton dose of 10^16^ cm^−2^ is shorter than that in the sample with the dose of 10^15^ cm^−2^. This means that the former coherence length should be shorter when compared to that of the latter, but in fact it is the opposite, which indicates that local superconductivity changes due to proton irradiation should also be considered as a cause of the reduction in the coherence length.

In order to understand the pinned vortex liquid phase under magnetic field in superconducting SmBCO-coated conductors, we would like to analyze the significantly broadening electrical resistivity resulting from the thermally activated flux flow for the vortex. According to TAFF theory, if the applied current density in the TAFF region is not large, the electrical resistivity is expressed as follows^26–29^:

1 $${\rm{\rho }}=(2{\rho }_{c}U/T)\exp (-U/T),$$

where $U={J}_{c0}BVL$ and ${\rho }_{c}={\nu }_{0}LB/{J}_{c0}$. ${\nu }_{0},\,L,\,B,\,{J}_{c0},\,V$ and *T* are the attempt frequency for the flux-bundle hopping, the hopping distance, the magnetic induction, the critical current density in the absence of flux hopping, the bundle volume and the temperature of sample, respectively. The detail definitions of *U* and ${\rho }_{c}$ indicate that $2{\rho }_{c}U/T$ is dependent on temperature and magnetic field. However, in many experimental studies of HTSCs, the prefactor $2{\rho }_{c}U/T$ was assumed as the temperature-independent constant ${\rho }_{0f}$. In this assumption, the relation for activation energy of $U(T,\,H)={U}_{0}(H)(1-t)$ is generally used, where $t=T/{T}_{c}$. The natural logarithm of Eq. (1) is

2 $$\mathrm{ln}\,\rho (T,\,H)=\,\mathrm{ln}\,{\rho }_{0}(H)-{U}_{0}(H)/T,$$

where $\mathrm{ln}\,{\rho }_{0}(H)=\,\mathrm{ln}\,{\rho }_{0f}+\,{U}_{0}/{T}_{c}$ and ${U}_{0}$ is the apparent activation energy for the flux flow. Differentiating Eq. (2) to the reciprocal of temperature, we obtain the equation of $\partial \,\mathrm{ln}\,\rho /\partial {T}^{-1}={U}_{0}(H)$, which indicates that the plot of $\mathrm{ln}\,{\rm{\rho }}$. vs. ${T}^{-1}$ should show a linear change in the TAFF region. Then its slope is ${U}_{0}$ and its *y*-axis-intercept is $\mathrm{ln}\,{\rho }_{0}(H)$. This is an Arrhenius relationship.

**Figure 7:**
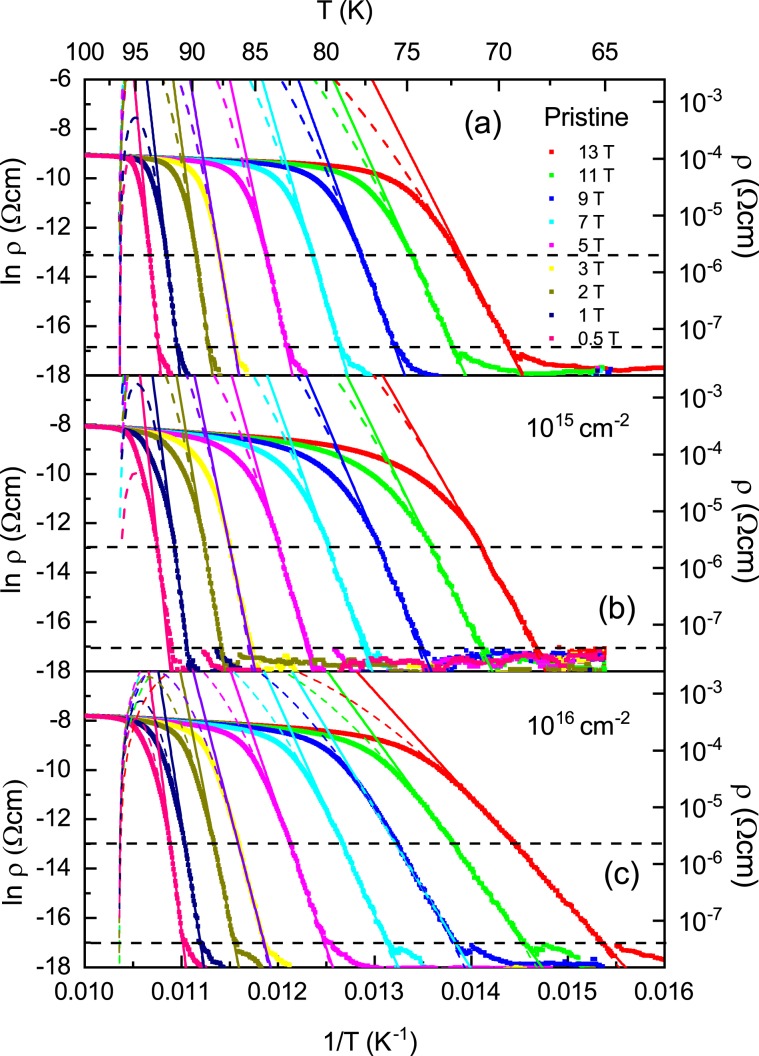
Data of $\mathrm{ln}\,{\rm{\rho }}$ vs. ${T}^{-1}$ obtained by measuring (scattered symbols) and data fitting $\mathrm{ln}\,{\rm{\rho }}$ vs. ${T}^{-1}$ using the activation energy equation $U(T,H)={U}_{0}(H){(1-t)}^{q}$ with *q* = 1 (dashed line) and 2 (solid line) in pristine (**a**), 10^15^ (**b**) and 10^16^ (**c**) cm^−2^ proton-irradiated samples. Horizontal black dashed lines are the boundary of the maximum and minimum electrical resistivities that satisfy the Arrhenius relation.

**Figure 8:**
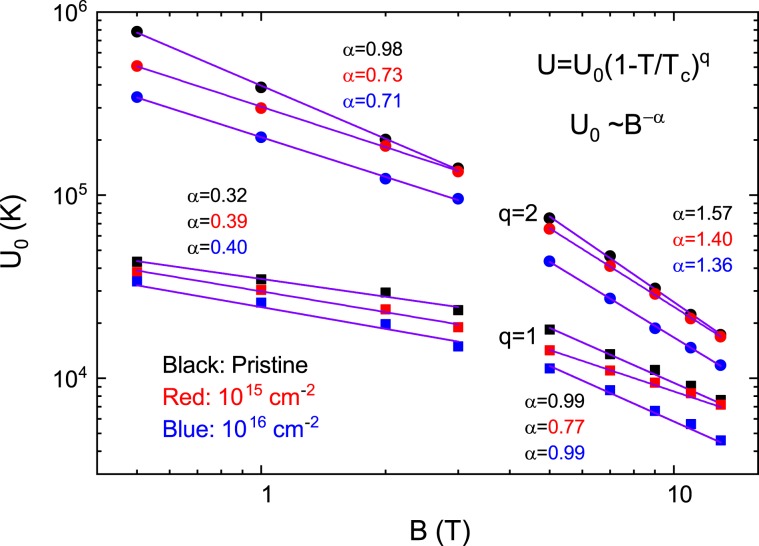
Log-log plot for ${U}_{0}(H)$ obtained when *q* = 1 (solid square symbol) and 2 (solid circle symbol) in the relationship of $U(T,H)={U}_{0}(H){(1-t)}^{q}$ in pristine (black scattered symbols), 10^15^ (red scattered symbols) and 10^16^ (blue scattered symbols) cm^−2^ proton-irradiated samples. Straight lines represent the linear fittings of the data.

The plots of $\mathrm{ln}\,{\rm{\rho }}$ vs. ${T}^{-1}$ for the pristine sample and the irradiated samples with the dose of 10^15^ and 10^16^ cm^−2^ are depicted in Fig. 7(a–c) to confirm the temperature region that satisfy the Arrhenius relationship. The data for each sample shows a fairly linear change in the temperature range corresponding to electrical resistivity values between 0.04 and 4 μΩcm, which are plotted in black dashed lines in Fig. 7(a–c). This temperature range is lled the TAFF temperature region. The determined TAFF regions for the pristine sample and the irradiated samples with the dose of 10^15^ and 10^16^ cm^−2^ are depicted in magenta color in Fig. 6(a–c), respectively. The temperature range between the upper temperature of the TAFF region and the ${T}_{k}$ is called TAFF critical region, which is an intermediate area from the unpinned vortex liquid state to the pinned vortex liquid state. If we draw linear regression on data with different magnetic fields in the TAFF temperature region, the slope is ${U}_{0}(H)$ and the *y*-axis-intercept is $\mathrm{ln}\,{\rho }_{0}(B)$. The linear regression for different magnetic fields is drawn with solid lines of different colors in Fig. 7(a–c). As shown in the figures, the linear regressions seem to agree well with the experimental results in the TAFF temperature region. Although not shown in the figure, the straight line extrapolated by the linear regression at each field passes almost one point of (1/*T*_*c*_, ln *ρ*_0*f*_). Here, *T*_*c*_ is 96.6 ± 0.5, 96.5 ± 0.3 and 96.5 ± 0.5 K and ln *ρ*_0*f*_ is 13.3 ± 0.5, 13.6 ± 0.5 and 6.3 ± 0.5 Ω∙cm for pristine sample and the irradiated samples with the dose of 10^15^ and 10^16^ cm^−2^, respectively. ${U}_{0}(H)$ determined by the regression is plotted on a log-log scale in Fig. 8. In the figure, ${U}_{0}(H)$ in each sample shows a linear change, which means ${U}_{0}(H)\, \sim \,{H}^{-\alpha }$, and its slope changes more steeply above 4 T. By irradiating the protons, the magnitude of ${U}_{0}(H)$ decreased in all magnetic fields compared to the pristine sample. Although proton-irradiated samples with many point defects caused by proton irradiation have many vortex pinning centers, the fact that ${U}_{0}(H)$ is smaller than that of the pristine sample suggests that the structure of the pinning center differs between pristine and proton-irradiated samples. The magnitude and *H*-dependence of ${U}_{0}(H)$ in the pristine sample are similar to those of an optimally doped-YBCO film^30^.

**Figure 9:**
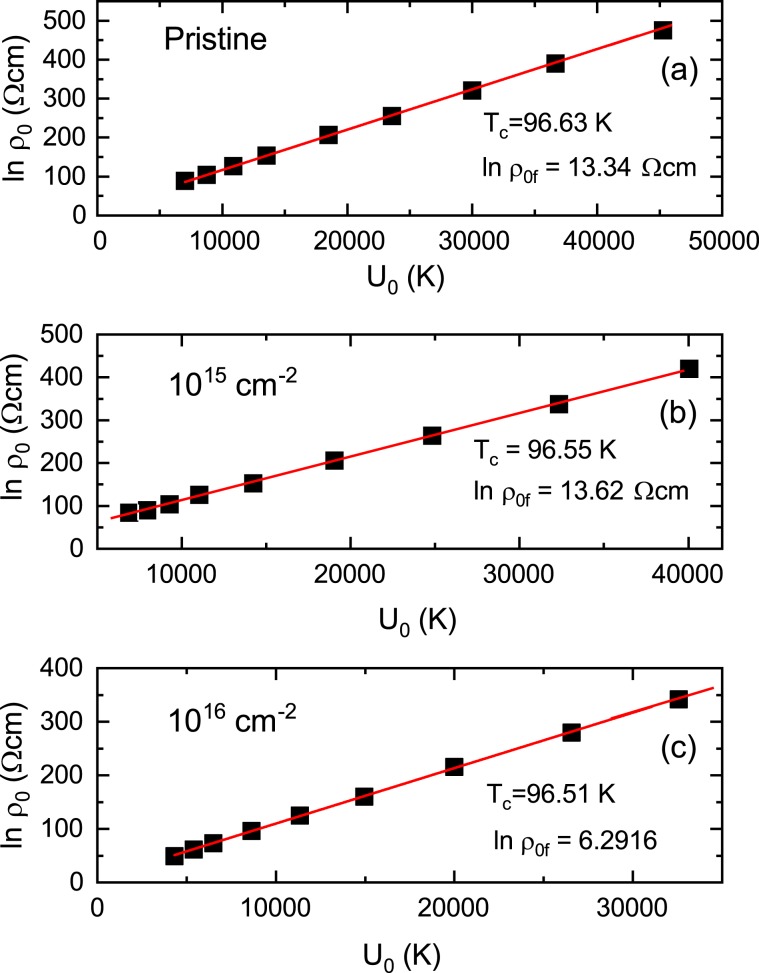
$\mathrm{ln}\,{\rho }_{0}({U}_{0})$ relations determined from Arrhenius plot in pristine (**a**), 10^15^ (**b**) and 10^16^ (**c**) cm^−2^ proton-irradiated samples as shown in Fig. 7. Solid lines in (**a**–**c**) show linear fits of the data.

The relationship of $\mathrm{ln}\,{\rho }_{0}({U}_{0})$ using the fitting parameters determined by the linear fit above is shown for the pristine sample and the irradiated samples with the dose of 10^15^ and 10^16^ cm^−2^ in Fig. 9(a–c), respectively. The relationship is well linearly-regressed using the formula $\mathrm{ln}\,{\rho }_{0}(H)=\,\mathrm{ln}\,{\rho }_{0f}+\,{U}_{0}/{T}_{c}$: As a result we obtain ${T}_{c}=96.63\,{\rm{K}}$, $\mathrm{ln}({\rho }_{0f})=13.34\,\Omega \cdot {\rm{cm}}$ for the pristine sample, ${T}_{c}=96.55\,{\rm{K}}$, $\mathrm{ln}({\rho }_{0f})=\,13.62\,\Omega \cdot {\rm{cm}}$ for the proton-irradiated sample with the dose of 10^15^ cm^−2^ and ${T}_{c}=96.51\,{\rm{K}}$, $\mathrm{ln}({\rho }_{0f})=6.29\,\Omega \cdot cm$ for that of 10^16^ cm^−2^. These values for each sample agree well with the values obtained by the coordinates of one point discussed above.

**Figure 10:**
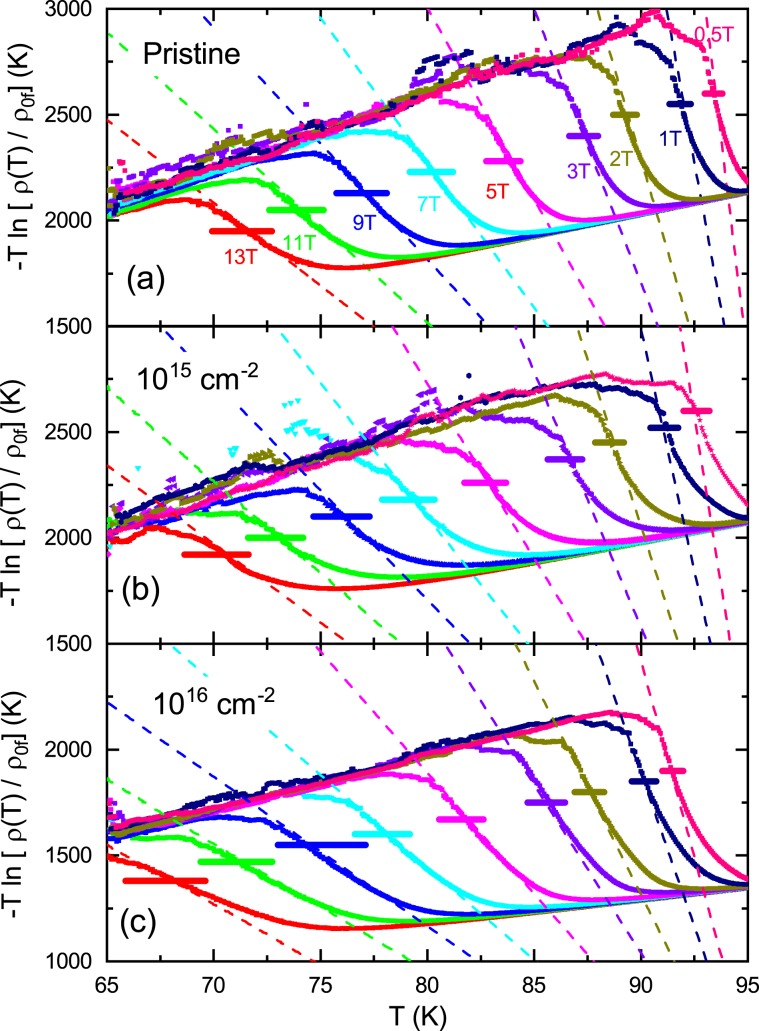
Different color symbols show $U(T,\,H)=-\,T\,\mathrm{ln}\,[\rho (T,\,H)/{\rho }_{0f}]$ and different color dashed lines show $U(T,H)=\,{U}_{0}(1-T/{T}_{c})$ for pristine (**a**), 10^15^ (**b**) and 10^16^ (**c**) cm^−2^ proton-irradiated samples. In (**a**–**c**), the thick horizontal lines of different colors show the TAFF area corresponding to different magnetic fields.

We need to verify the accuracy of ${U}_{0}$ obtained from the indirect approach by the Arrhenius assumption discussed above. From the Arrhenius equation (Eq. (2)), the temperature dependence of the activation energy at each field is given by $U(T,H)=-\,T\,\mathrm{ln}\,[\rho (T,\,H)/{\rho }_{0f}]$, which can be obtained directly from the measurement data and is shown for the pristine sample and the irradiated samples with the dose of 10^15^ and 10^16^ cm^−2^ in Fig. 10(a–c), respectively. In the calculations, we used ${\rho }_{0f}$ and ${T}_{c}$ determined in Fig. 9(a–c). As shown in the figures, each sample shows a similar temperature dependence: as the temperature decreases from high temperature, the magnitude of $U(T,\,H)$ decreases almost linearly, then increases steeply and then decreases again. The TAFF region determined earlier, which has a different temperature range depending on the magnetic field, is also drawn with thick horizontal lines at the middle height of Fig. 10(a–c). In the figure, data of $U(T,\,H)$. and thick horizontal lines with the same magnitudef magnetic field are displayed in the same color. The temperature range in which $U(T,\,H)$ increases rapidly as the temperature decreases corresponds to the TAFF region. On the other hand, the temperature dependence of the activation energy on the flux flow used in the Arrhenius assumption is given by $U(T,H)={U}_{0}(1-T/{T}_{c})$. We calculated $U(T,\,H)$ using the ${U}_{0}$ and ${T}_{c}$ obtained earlier and plotted them using dashed lines with different colors according to the magnitude of the magnetic field in Fig. 10(a–c). As shown in the figures, the agreement between the data of $-T\,\mathrm{ln}\,\rho (T,H)/{\rho }_{0f}$ and $\,{U}_{0}(1-T/{T}_{c})$ in the TAFF region at first glance is good for all measured magnetic fields. In detail, the two lines intersect weakly in the TAFF temperature range, like the tilted “chi” of a Greek letter. This is the limitation of the Arrhenius relationship.

To investigate where the deviation came from, we plotted the temperature dependence of $\partial \,\mathrm{ln}\,\rho /\partial {T}^{-1}={U}_{0}(H)$ obtained by differentiating Eq. (2) in Fig. 4(a–c) for the pristine sample and the irradiated samples with the dose of 10^15^ and 10^16^ cm^−2^, respectively. As shown in the graph, $\partial \,\mathrm{ln}\,\rho /\partial {T}^{-1}={U}_{0}(H)$ increases as the temperature decreases in the TAFF temperature range, and then fluctuates significantly in the temperature region below the TAFF.

Let us first discuss why ${U}_{0}$ fluctuates significantly in the temperature region below the TAFF. It is physically meaningless data due to a signal smaller than the minimum signal that can be detected in electrical resistivity measurements. Thus it is not known here whether the vortex glass-liquid transition occurs below the TAFF temperature. Note that vortex glass-liquid transitions rapidly increase ${U}_{0}$ at temperatures lower than the TAFF temperature range^20,31^.

**Figure 11:**
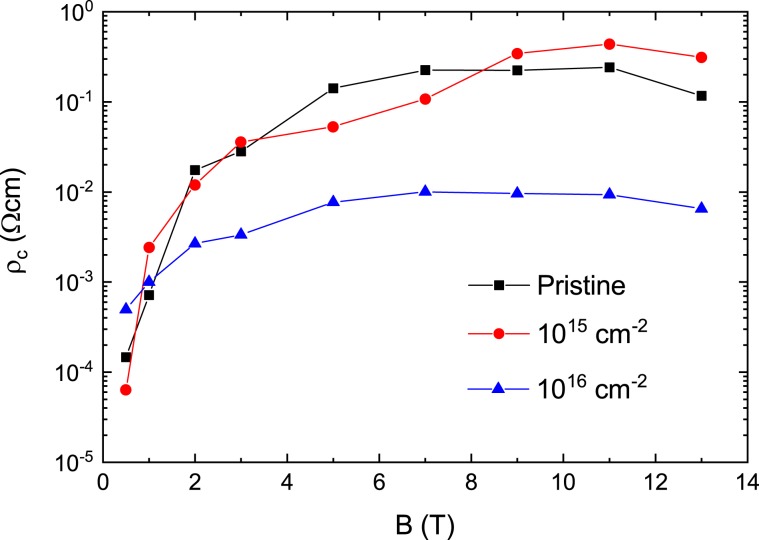
The parameter ${\rho }_{c}(H)$ obtained by fitting the data of $\mathrm{ln}\,{\rm{\rho }}$ vs. ${T}^{-1}$ using Eq. (3) in the text is plotted for pristine, 10^15^ and 10^16^ cm^−2^ proton-irradiated samples.

Next, discuss the temperature dependence of ${U}_{0}$ in the TAFF temperature range. This is clearly different from the Arrhenius relationship, assuming that the prefactor is constant with temperature changes. The temperature dependence of ${U}_{0}$ is the cause of the slight discrepancy in Fig. 10(a–c) discussed earlier. The temperature dependence of ${U}_{0}$ results in a temperature dependency of prefactor $2{\rho }_{c}U/T$. We try to obtain ${U}_{0}$ more accurately than the Arrhenius relationship, taking into account the temperature dependence of the prefactors. This result helps to understand the vortex movement more accurately. Actually, in order to consider the temperature dependence of the prefactor $2{\rho }_{c}U/T$, the introduction of the nonlinear relationship of $U(T,\,H)$ vs. *T* has been performed in many cuprates and iron-based superconductors^23,29,30,32^. Using the nonlinear relationship $U(T,H)={U}_{0}(H){(1-t)}^{q}$ in Eq. (1),

3 $$\mathrm{ln}\,\rho =\,\mathrm{ln}(2{\rho }_{c}{U}_{0})+q\,\mathrm{ln}(1-t)-\,\mathrm{ln}\,T-{U}_{0}{(1-t)}^{q}/T$$

and

4 $$-\frac{\partial \,\mathrm{ln}\,\rho }{\partial {T}^{-1}}=[{U}_{0}{(1-t)}^{q}-T]\,[1+qt/(1-t)]$$

are derived. Here, ${\rho }_{c}$ and ${U}_{0}$ do not depend on the temperature, and ${T}_{c}$ is obtained from the Arrhenius relation. The values of ${T}_{c}$ obtained by the Arrhenius relationship show good agreement with those of ${T}_{c}$ in Fig. 2. Therefore, it is appropriate to use these values as the value of ${T}_{c}$ in the above two equations. When performing the regression of the experimental data with the Eqs. (3) and (4) below, it is important to know ${T}_{c}$ in advance because the fitting parameter *q* is the exponential form of ${T}_{c}$ in the above two equations; otherwise, the value of the fitting parameter obtained by the regression will be physically meaningless. The data of $\mathrm{ln}\,\rho$ vs. ${T}^{-1}$ and $-\partial \,\mathrm{ln}\,\rho /\partial {T}^{-1}$ calculated from the measured ${\rm{\rho }}(T)$ in the pristine sample and the irradiated samples with the dose of 10^15^ and 10^16^ cm^−2^ are plotted using scattered symbols with different colors according to the magnitude of the magnetic field in Figs. 7(a–c) and 4(a–c), respectively. As shown in the figure, the data of $\mathrm{ln}\,\rho$ vs. ${T}^{-1}$ and $-\partial \,\mathrm{ln}\,\rho /\partial {T}^{-1}$ are well reproduced in the TAFF temperature region using Eqs. (3) and (4) with *q* = 2, respectively. The reproduced data are plotted using dashed lines with different colors according to the magnitude of the magnetic field in Fig. 7(a–c) and 4(a–c). The fitting parameters ${U}_{0}$ obtained in the two fittings show good agreement with each other. The ${U}_{0}(H)$ and ${\rho }_{c}(H)$ determined from the regression are plotted in Figs. 8 and 11, respectively. The magnitude of ${U}_{0}$ was reduced by proton irradiation, which is similar to the results obtained by the Arrhenius relationship. Therefore, it is resonalbe to think that the decrease in ${U}_{0}$ is due to the aforementioned reasons. The thermally activated energy ${U}_{0}(H)$ exhibits a similar power law dependence with different exponents α in low and high magnetic fields: α = 0.99 for ${\mu }_{0}H < 3\,{\rm{T}}$ and α = 1.57 for ${\mu }_{0}H > 4\,{\rm{T}}$ for pristine sample and α = ~0.74 for ${\mu }_{0}H < 3\,{\rm{T}}$ and α = ~ 1.38 for ${\mu }_{0}H > 4\,{\rm{T}}$ for both proton-irradiated samples. This indicates that ${U}_{0}(H)$ decreases slowly when increasing magnetic fields in the proton-irradiated samples as compared to the pristine sample, which means that an appropriate amount of irradiated protons on the sample can be one of the methods that favor high magnetic field applications. Compared with the results obtained in the Arrhenius relationship, the ${U}_{0}(H)$ obtained by this method in each sample is as high as one order, but the magnetic field dependence of ${U}_{0}(H)$ decreases more strongly.

Let’s discuss the *H*-dependency of ${U}_{0}$. The reduction of ${U}_{0}$ in the form of ${U}_{0}(H) \sim {H}^{-\alpha }$ in each sample cannot be explained by the collective elastic creep theory because the theory shows an increase in ${U}_{0}$ at high magnetic fields^26,33^. So, we can think that the *H*-dependence of ${U}_{0}$ in the proton-irradiated samples in the low magnetic field region is related to the plastic deformation and entanglement of the vortex caused by point defects in the weakly pinned vortex-liquid phase. According to the plastic flux creep theory^22,34,35^, the activation energy due to the plastic deformation and the entanglement of vortex lines decreases in the form of ${U}_{0} \sim {H}^{-0.5}$. In such entangled vortex liquids, the relative motion to each other is significantly modified by the cutting and recombination of the vortex line, so that the magnitude of the exponent ${\rm{\alpha }}$ can be changed around 0.5. In fact, α = 0.7 was observed in untwinned YBCO single crystals irradiated with proton^21^. A faster decay of ${U}_{0}(H)$ can be suggested due strongly pinned or entangled vortex liquids. In this respect, point defects produced by proton irradiation in proton-irradiated samples play an important role in vortex pinning; in magnetic fields below 4 T, point defects lead to weakly pinned and entangled vortex liquids, while higher magnetic fields lead to strongly pinned and entangled vortex liquids. This pinning change causes ${U}_{0}(H)$ to be bent around 4 T, as shown in Fig. 9. The bending in ${U}_{0}(H)$ was not observed in the proton-irradiated YBCO^21^.

On the other hand, ${U}_{0}(H)$ shows approximately 1/*H* dependence in the pristine sample. SmBCO coated conductors fabricated in a similar manner have been reported to have correlated disorders such as dislocations and splayed extended-c-axis defects^36^. According to papers reported previously, the dislocations in all directions stabilize the Bose glass phase at low temperatures and the vortex lines are disentangled in the vortex liquid state seen in higher temperature ranges, which causes a dependency of *U*_0_ ~ 1/*H*^20,37–41^. On the other hand, correlated disorders caused by splayed extended-c-axis defects stabilize the splayed glass phase at low temperatures and entangle the vortex lines in the vortex liquid state at relatively high temperatures^20,37^. This also causes a dependency of *U*_0_ ~ 1/*H*^20^. As discussed earlier, in our experiments, no vortex glass phase was observed that would exist at low temperatures below the vortex liquid state. However, as mentioned above, the correlated disorders caused by dislocations and splayed extended-c-axis defects expected to be present in the film are believed to exhibit the dependence of *U*_0_ ~ 1/*H*. This is strongly supported by results showing a larger ${U}_{0}$ in the pristine sample compared to the proton-irradiated sample, because ${U}_{0}$ due to correlated disorder is larger than that due to point defects under the same conditions^20^. Although proton-irradiated samples with many point defects resulting from proton irradiation have many vortex-pinning centers, showing smaller ${U}_{0}$ in proton-irradiated samples means that the structure of the pinning center differs between the pristine and proton-irradiated samples.

In the high magnetic field of the pristine sample, ${U}_{0}$ decreases rapidly as *H* increases compared to the low magnetic field. If the vortex lines are more strongly pinned to splayed extended-c-axis defects under high magnetic fields and the vortex lines become more entangled, the vortex lines are more easily cut and recombined. Then, the relative motion of vortex lines to each other will be significantly modified to reduce ${U}_{0}$ more rapidly, which can be thought of as similar to the entanglement of vortex lines due to point defects in the proton irradiated sample discussed above.

As a result, the main pinning of the pristine sample was caused by correlated disorder, and the ${U}_{0}$ was relatively large. In contrast, the main pinning of the sample in which the protons were irradiated was due to the point defect and the ${U}_{0}$ was relatively small. Based on this, the flux and energy used in proton irradiation produced a local annealing effect on the sample and it played a role in eliminating correlated disorders. In the proton irradiation experiment, although the method of preventing temperature rise during proton irradiation was adopted as discussed in the method section later in the paper, the local annealing effect could not be excluded.

## Conclusion

To investigate changes in vortex pinning pattern caused by proton irradiation from SmBCO superconducting tapes, the in-plane electrical resistivity was measured near *T*_*c*_ under various applied magnetic field with *H*//*c*-axis. Though *T*_*c*_ at *H* = 0 hardly changed before and after the proton irradiation, the magnitude of the electrical resistivity in the normal state showed a pronounced increase by newly created point-defects due to proton irradiation. The electrical resistivity near *T*_*c*_ was broadened with the increase of the magnetic field. The broadening was well interpreted by a thermally activated flux flow model: the temperature dependence of activation energy shows $U(T,H)={U}_{0}(H){(1-T/{T}_{c})}^{q}$ with the same *q* = 2 in the three samples and the field dependence of activation energy shows a two-step ${U}_{0} \sim {H}^{-\alpha }$ with larger exponents above 4 T. It may conclude that the main cause of the field dependence is due to the disentangled or/and entangled vortex liquid caused by the pinning with the correlated disorders present in the pristine sample and the plastic deformation and vortex entanglement caused by the pinning with artificially created point defects as a result of the proton irradiation in the irradiated samples.

## Methods

High quality SmBCO coated conductor tapes were deposited on IBAD-MgO substrates by evaporation using a drum in dual chambers at the Korea Electrotechnology Research Institute (KERI)^8^. The system is a reactive co-evaporation system used for deposition of coated conductors^12^. The structure of SmBCO films is Ag (3 μm)/SmBCO (5 μm)/LaMnO_3_ (20 nm)/epi-MgO (20 nm)/IBAD-MgO (10 nm)/Y_2_O_3_ (10 nm)/Al_2_O_3_ (40 nm)/Hastelloy (80 μm).

Proton irradiation was performed at 300 K using an MC-50 cyclotron installed at KIRAM (Korea institute of radiological & medical sciences). The proton energy used is 30 MeV and two specimens with a total dose of 1 × 10^15^ and 1 × 10^16^ cm^−2^ were produced. To prevent the temperature-rise of the specimen as much as possible during the proton irradiation, a weak flux density rate of ~2 × 10^11^ cm^−2^∙s was used and the specimen was attached to a copper water block.

The x-ray diffraction analysis was performed on a PANalytical X-ray powder diffractometer with the Bragg-Brentano geometry on film-type samples using Cu Kα radiation (40 kV, 30 mA and λ=1.5406 Å) with step size of 0.026° (2θ) and scan rate of 0.78°/min.

The in-plane electrical resistivity for *H*//*c* in SmBCO coated conductor tapes were measured by using a 16 T Oxford superconducting system. A four-point collinear probe method is used in the experiment. The resolution of the resistivity measurement is a few 10^−8^ Ω∙cm. The temperature dependence of the electrical resistivity was measured in detail at a temperature interval of about 0.1 K in a wide range of magnetic fields of μ_0_*H* = 0, 0.5, 1, 2, 3, 5, 7, 9, 11 and 13 T.
